# Therapeutic peptides of *Mucuna pruriens* L.: Anti‐genotoxic molecules against human hepatocellular carcinoma and hepatitis C virus

**DOI:** 10.1002/fsn3.2248

**Published:** 2021-03-25

**Authors:** Seyedeh Faezeh Taghizadeh, Majid Azizi, Javad Asili, Fatemeh Sadat Madarshahi, Hasan Rakhshandeh, Yoshiharu Fujii

**Affiliations:** ^1^ Pharmaceutical Research Center Pharmaceutical Technology Institute Mashhad University of Medical Sciences Mashhad Iran; ^2^ Department of Horticultural Science Ferdowsi University of Mashhad Mashhad Iran; ^3^ Department of Pharmacognosy Faculty of Pharmacy Mashhad University of Medical sciences Mashhad Iran; ^4^ Department of Horticultural Science Faculty of Agriculture Islamic Azad University of Shirvan Shirvan Iran; ^5^ Pharmacological Research Center of Medicinal Plants Mashhad University of Medical Sciences Mashhad Iran; ^6^ Department of International Environmental and Agricultural Sciences Tokyo University of Agriculture and Technology Fuchu Japan

**Keywords:** beans, DNA damage, herbal therapy, liver cancer, mutation

## Abstract

To assist the development of new therapeutic strategies for several disorders, biologically active peptides/proteins obtained from plant sources can be considered. Current study expected to determine the biological activities of peptide fractions of *Mucuna pruriens* against hepatocellular carcinoma cell lines (HepG2/ADM, HepG2, SMMC‐7721, and QGY‐7703), as well as normal cell line to prove their selectivity. Moreover, anti‐genotoxicity and antiviral activity against the hepatitis C virus (HCV) were assessed. The methods of this study were to isolate the peptides of *M. pruriens* and hydrolysate fractionation via fractionated pepsin‐pancreatin hydrolysates by ultrafiltration/high‐performance ultrafiltration cell, identify anti‐hepatoma activity of peptide fractions human liver cancer and normal cells by (3‐(4,5‐dimethylthiazole‐2‐yl)‐2,5‐biphenyl tetrazolium bromide) (MTT) assay, determine anti‐*HCV*, and assess anti‐genotoxic effect of peptide fractions against damage that induced via alkylating agent methyl methanesulphonate in human mononuclear cells. The results showed that the fraction 5–10 kDa has been reported to exhibit significant cytotoxic activity against HepG2 and QGY‐7703. It was proven that both of 5–10 and >10 kDa fractions are active against *HCV*. The cytotoxic concentration (CC_50_) of 5–10 kDa against the cell line was 703.04 ± 5.21 µg/ml. Anti‐genotoxic activities of the peptide fractions were evaluated as mean values for the analyzed comet images. In this regard, the highest activity of protecting DNA damages was observed by the peptide fraction of 5–10 kDa. This study revealed the potential ability of peptide fractions of *M. pruriens* for the treatment of liver cancer, HCV, and high activities of protecting DNA damages.

## Graphical abstract

This study revealed the potential ability of peptide fractions of *M. pruriens* for the treatment of liver cancer, hepatitis C virus and high activities of protecting DNA damages.

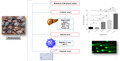

## 1 INTRODUCTION

Hepatocellular carcinoma (HCC) with more than 700,000 deaths annually is the second leading cancer worldwide. Despite advances in medical, current approaches to the management of hepatocellular including locoregional and surgical therapies, radiotherapy, and chemotherapy are restricted due to the liver traits. Cytotoxic chemotherapy with lipiodol, cisplatin, and doxorubicin has revealed moderate efficacy with respect to several severe effects. Considering the value of the novel/more efficient chemotherapeutic agents for the treatment of HCC, various studies has been done to evaluate the novel substitute from natural sources for the treatment of several infectious and chronic diseases (Hartke et al., 2017; Wang et al., 2018). Hepatitis C virus (*HCV*) is becoming a serious infection source liver disorders in millions of people every year. Most of the infected populations are posed to risk cirrhosis. It can cause major effects on the doubling of HCC. Hepatitis C virus as an RNA virus is categorized in the genus*Hepacivirus* belonging to the Flaviviridae family (Khachatoorian et al., 2015). Despite successful development in drugs against *HCV*, viral resistance, prospective undesirable risk, and high cost of antiviral drugs are still considered restrictions to improve the antiviral agents. Thus, it is still necessary to develop the novel agents that aim various phases of the viral life cycle (Taghizadeh et al., 2019; Taghizadeh, Rezaee, Mehmandoust, Madarshahi, et al., 2019).

The natural products showed extensive spectrum of biological traits (Amini et al., 2014; Heidari et al., 2014). Several peptides including proteinase and glycosidase inhibitors, as well as cyclic peptides, are produced by plants. These therapeutic peptides have been found in roots, stems, leaves, and flowers of a few species from the Cucurbitaceae, Fabaceae, Rubiaceae, Solanaceae, and Violaceae families (Aminifard et al., 2012; Craik et al., 2018). Several proteins such as Mistletoe Lectin‐I and Bowman‐Birk protease inhibitor are currently under clinical trials against malignant melanoma, prostate cancer, and oral carcinomas (Wani et al., 2019). It was previously shown that Vitri A peptide possesses broad anticancer properties. Its cytotoxicity results showed the IC_50_ values ranging from 0.6 to 6.03 µg/ml for U‐937GTB, U251 MDA‐MB‐231, A549, DU145, BEL‐7402 cell lines (Tang et al., 2010). Furthermore, Pis v 1 and Pis v 2.0101 isolated from pistachio (*Pistacia vera* L.) showed significantly cytotoxic activities against human ovarian carcinoma (A2780), human colon carcinoma (HTC), human glioblastoma (U‐87‐MG), human breast adenocarcinoma (MCF‐7), and human prostate cell lines (PC3 and DU‐145) (Taghizadeh, Rezaee, Mehmandoust, Badibostan, et al., 2019; Taghizadeh, Rezaee, Mehmandoust, Madarshahi, et al., 2019). The antiviral trait of Beetin 27 (BE27) from *Beta vulgaris* L. leaves was demonstrated. This defense protein has been attributed to its RNA and DNA polynucleotides. BE27 exhibited superoxide dismutase activity and produced the signal compound hydrogen peroxide (Iglesias et al., 2015). Furthermore, lipopeptide based on viral fusion inhibitors such as cholesterylated peptide *HIV*‐1/2 fusion inhibitors with effective and long‐lasting antiviral activity can also be used as novel treatment for clinical development (Zhu et al., 2019). Different herbal compounds with biologically activities are of limited therapeutic use due to their carcinogenic, toxicological, and mutagenic properties. Therefore, the genotoxic analysis seems to be necessary for their potential use as a new therapeutic agent. Bio anti‐mutagens can inactive the mutagenic mechanisms as well as the effect on DNA damage repair over decrease in mutation rate (Goswami et al., 2019). Méndez‐Espinoza et al. reported that the anti‐genotoxic activity against direct mutagens was observed in *Cantharellus cibarius* (Mendez‐Espinoza et al., 2013).

*Mucuna pruriens* belongs to Fabaceae family is used worldwide in complementary medicine. This legume found in India and China. It has immuno‐modulatory potential with significant improvement in immunoglobulin levels. It is also found that several amino acids as well as proteins including globulins and albumins can be associated with therapeutic properties (Rai et al., 2017; Ulu et al., 2018). Researches have shown the several biological traits of *M. pruriens* (Yadav et al., 2015), but there is no report in the literature on the beneficial effects of peptides isolates from *M. pruriens*. In the current study, we tried to provide the practical tools to promote therapeutic peptides usages with a particular focus on liver cancer and HCV. Due to the fact that we emphasized on peptide fractions derived from *M. pruriens* to examine whether they can provide the prediction performance of cytotoxicity and antiviral activity. Current study expected to (a) determine the biological activities of peptide fractions of *M. pruriens* against HCC cell lines (HepG2/ADM, HepG2, SMMC‐7721, and QGY‐7703), as well as normal cell line; (b) evaluate anti‐genotoxicity; and (c) assess the antiviral activity of the peptide fractions against the HCV.

## 2 MATERIAL AND METHODS

### 2.1 Plant material

*Mucuna pruriens* beans were deposited at the herbarium of Mashhad University of Medical Sciences, Mashhad Iran, and were cultured in the research greenhouse of Ferdowsi University of Mashhad, Iran (latitude 36°16′N, longitude 59°36′E, and 985 m altitude). The beans were washed two times and dried at room temperature (25°C). They were packed in glass containers, kept at −20°C, and protected from light before the analysis.

### 2.2 Chemicals

The analytical grade reagents were supplied by Merck. Pepsin (CAS No. 9001‐75‐6), pancreatin (CAS No. 8049‐47‐6), Cisplatin (CAS No. 15663‐27‐1), dimethyl sulfoxide (DMSO) (CAS No. 67‐68‐5), and MTT (CAS No. 298‐93‐1) were purchased from Sigma‐Aldrich (Steinheim, Germany). Dulbecco Modified Eagle Medium (DMEM) (CAS No. 10‐013‐LX), ethylene diamine tetric acid (EDTA) (CAS No. 46‐034‐CI), penicillin‐ streptomycin (CAS No. 30‐002‐CI), and Fetal Bovine Serum (FBS) (CAS No. 35‐077‐CI) were procured Gibco, BRL.

### 2.3 Sample preparation

*Mucuna pruriens* beans were ground with an electric mill (Toos Shekan, Khorasan, Iran). In order to remove the smallest particles, they filtered through a 2‐mm mesh size sieve. A fluidizing air bed was used to separate the hull beans and then the flour obtained was easily milled.

### 2.4 Protein concentration

Protein concentration was conducted based on earlier described by Herrera Chalé et al., (2014). 1.0 kg *M. pruriens* bean flour was extracted for 1 hr using 3% sodium bisulfite (1:10 [w:v] ratio, pH 8). In order to isolate the fiber solids from the protein and the starch‐containing liquid portion, the extract was passed through a 0.177‐mm mesh size sieve. The remaining solids were washed three times using 300 ml of 3% sodium bisulfite. The digested sample was remained 30 min to separate the starch and solubilized protein. The final pH of the protein suspension was adjusted to 4.2 using 1.0 MHCl. Finally, the suspension was centrifuged at 1,317 g and freeze‐dried at −47°C for 20 min (Chalé et al., 2014).

### 2.5 Hydrolysis of the protein isolates

Enzymatic hydrolysis was performed in a 1,000‐ml reaction vessel equipped with a stirrer. The treatments were as follows: pepsin (Sigma, P7000‐ 100G) and pancreatin (Sigma, P3292‐100G). In order to prepare a protein solution, the protein isolates were suspended in distilled water (10% w/v). Then, pepsin‐pancreatin system was added to the solution for 90 min (each of them was incubated for 45 min). The hydrolysis parameters were substrate concentration 4%; enzyme/substrate ratio 1:10. The pH was adjusted to 7.5 and 2.0, for pancreatin and pepsin, respectively, using 1 mol/L sodium hydroxide. The temperature was held at 37°C for analyses. The enzymatic hydrolysis was stopped by increasing the temperature to 80°C for 20 min. In order to eliminate the insoluble portion, the digested sample was centrifuged at 1,317 g for 20 min (Martínez‐Leo et al., 2019).

### 2.6 Degree of hydrolysis (DH) percent

Degree of hydrolysis (%) was measured via the following Equation (Martínez‐Leo et al., 2019):

(1) $$\text{DH (\textbackslash{}\% )}=\frac{\text{Number of hydrolysed bonds (HBs)}}{\text{Total number of peptide bonds (PBs) per protein equivalent (Peq)}}\times 100$$

The total number of PBs per Peq is due to the amino acid composition of the raw material (7.66 mmol/g of protein for *M. pruriens*). All experiments were done in triplicate. Martínez‐Leo et al. reported the amino acid components of *M. pruriens* peptides (Martínez‐Leo et al., 2019).

### 2.7 Hydrolysate fractionation

Hydrolysate fractionation was done via a little modified method that was described by Cho et al. Ultrafiltration/high‐performance ultrafiltration cell (Millipore Inc.) was used for pepsin‐pancreatin hydrolysates fractionation. Several molecular weights cut off membranes including 1, 3, 5, and 10 kDa were used for preparing the five fractions. The hydrolysate process was started with 10 kDa as the largest cartridge and ended with 1 kDa. Therefore, five peptide fractions were as follows: <1, 1–3, 3–5, 5–10, and >10 kDa. All the fractions were freeze‐dried and kept at −20°C until biological analysis (Chalé et al., 2014).

### 2.8 Cytotoxic assay

#### 2.8.1 Cell culture

The human hepatoma cell lines including HepG2/ADM, HepG2, SMMC‐7721, QGY‐7703, normal cell line mouse embryonic fibroblast cells NIH 3T3, and cisplatin were maintained in DMEM supplemented with 10% (v/v) FBS, penicillin, and streptomycin 100 U/ml. The cells were cultured in 5% CO_2_ incubator at 37°C. In order to maintain the multidrug‐resistant characteristics of the cells, cisplatin (1.2 μM) was added to the culture medium (Shakeri et al., 2019).

#### 2.8.2 Cell viability

The cells viability was tested by MTT test. The cells (1 × 10^4^/well) were seeded. Each well contains 100 µl DMEM medium and 10% FBS. After 24 hr, serial dilutions of the peptide fractions and cisplatin (positive control) were added. 100 μl of MTT (5 mg/ml in DMEM) was added for 4 hr. After this time, the medium with MTT was removed. Since, to dissolve the formazan crystals, 150 μl DMSO was added. The absorbance was determined by a spectrophotometric microtiter plate reader at 590 nm (Taghizadeh, Rezaee, Mehmandoust, Badibostan, et al., 2019; Taghizadeh, Rezaee, Mehmandoust, Madarshahi, et al., 2019). The minimum inhibition concentration for inhibiting 50% of the viable cells (IC_50_) was calculated via the relative viability over the peptide fractions concentration curve (Shakeri et al., 2017).

### 2.9 Antiviral assay

The *HCV* was propagated in HEp‐2 cells. HEp‐2 cells were preserved in the condition described above. The virus titer was performed via 50% Tissue Culture Infectious Dose (TCID_50_) on HEp‐2 cells, and then they were kept at −80°C. The antiviral effect of peptide fractions was evaluated due to cell death in HEp‐2 and inhibitory activity on virus‐induced cytopathogenicity. The HEp‐2 cells were seeded at 10^4^ cells/well with 5% CO_2_ (at 37°C). The culture media was removed (after 24 hr). The cells were washed with PBS, then 100 μl of each peptide fractions was mixed with DMEM 2% supplemented with 10% FBS. They were inoculated with 50 μl of 2% DMEM and 100 TCID_50_ of *HCV*. Thus, the virus inhibition percentage was calculated as follows (Snene et al., 2017):

(2) $$\text{Virus inhibition (VI)(}\%)=\frac{\left(T‐\text{Vc}\right)}{\left(\text{Cc}‐\text{Vc}\right)}\times 100$$

T: Optical Density of cells treated with peptide fractions

Vc: Optical Density of virus control

Cc: Optical Density of cell control

The antiviral effect curve plotted via VI (%) against peptide fractions levels. The Selectivity Index (SI) was defined as follows (Taghizadeh et al., 2018).

(3) $$\text{SI}=\frac{\text{CC}50}{\text{IC}50}$$

IC_50_: 50% of cytopathic effect

CC_50_: 50% inhibition of normal cells growth

### 2.10 Genotoxic assay

#### 2.10.1 HMNCs isolate

HMNCs isolation was performed based on the method described by Méndez‐Espinoza et al. Blood samples of three healthy males (22, 26, and 33 years old) were selected for HMNCs isolate. Venipuncture with EDTA solution was used to take the blood samples. Purification of HMNCs was assayed through centrifugation via a Histopaque‐1077. The viability percentage was determined by trypan blue solution by 0.4%. The viable cell level was adjusted to 200,000 HMNCs/ml (Park et al., 2002).

#### 2.10.2 DNA damage

To obtain 90% viability in cell cultures, various concentrations of MMS were analyzed by a hemocytometer and the trypan blue exclusion dye at 0.4%. Eventually, MMS concentration was adjusted to 35 mM (Mendez‐Espinoza et al., 2013).

#### 2.10.3 Comet assay

The comet assay was conducted via the alkaline version with high sensitivity. It was easy to identify breaks in apurinic/apyrimidinic (AP) sites, open sites, and single‐strand DNA. To cover the glass slides with a thin agarose layer, 180 µl of 0.5% regular agarose, 75 µl of 0.5% agarose, 75 µl low melting point (LMA), and 5 µl HMNCs mix was used; and storing on an ice bath for 5 min to solidify. In order to protect cells, the slides were covered again with a second layer of agarose with a low melting point. The slides were absorbed in lysis buffer including 100 mM EDTA, 1% Triton, 10 mM Tris, 10% dimethyl sulfoxide, and 2.5 mM sodium chloride for 24 hr at 4°C in the dark. Then to unwind the DNA, the slides were moved to a solution (10 mM Na_2_EDTA, 300 mM sodium hydroxide, pH 13) for 20 min. The slides were immediately placed into electrophoresis apparatus for 20 min at 25 V (300 mA); followed by rinsing with Trisbase solution (0.4 M, pH 7.5) for 5 min. The following microscopic evaluation with an epifluorescent microscope (Axiostar Plus) was carried out. The mean tail length (µm) ± *SD* formed by the DNA fragments of 100 individual cells was calculated for each treatment (Martínez‐Leo et al., 2019).

### 2.11 Experimental design

The protocol included peptide fractions, positive control (HMNC + 35 mM MMS for 2 hr at 37°C), and negative control (HMNC). The peptide fractions (HMNC + 0.2% peptide fraction) were evaluated for anti‐genotoxic assay. Each treatment consisted of 300 cells. DNA damage reduction (DNA DR) (%) was calculated using Equation 4 (Mendez‐Espinoza et al., 2013).

(4) $$\text{DNA DR(}\%)=\frac{\text{Mean A}‐\text{Mean B}}{\text{Mean A}‐\text{Mean C}}\times 100$$

A: Positive control

B: Treatment

C: Negative control

### 2.12 Statistical analysis

These experiments used as a random block design in triplicates. The data were presented as mean ± standard deviation (*SD*). Variance analysis was carried out by SPSS software version 16.0, followed by *posthoc* Tukey test was performed to the comparison way. Mean values were calculated significantly different at *p* ˂ .05.

## 3 RESULTS

### 3.1 Protein content and DH of *M. pruriens*

**FIGURE 1:**
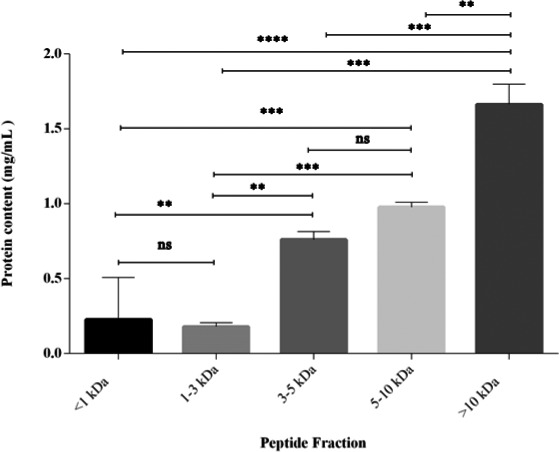
Protein content of *M. pruriens*

Based on the results, *M. pruriens* protein concentrate contained 67.11 ± 5.1% protein, whether, the protein contents of peptide fractions were 1.53 ± 0.24, 0.94 ± 0.20, 0.70 ± 0.12, 0.15 ± 0.05, and 0.063 ± 0.001 mg/ml in the fractions > 10, 5–10, 3–5, 1–3, and <1, respectively (Figure 1). The DH index of sequential pepsin–pancreatin hydrolysis was 32.51 ± 3.60% at 90 min.

### 3.2 Cytotoxic effects of peptide fractions

The IC_50_ values of peptide fractions against different cancerous cell lines were shown in Table 1. Among five peptide fractions, 5–10 kDa exhibited significant growth inhibitory effects against human HCC. It should also be noted that the fraction 5–10 kDa has been reported to exhibit significant cytotoxic activity against HepG2 and QGY‐7703 (IC_50_ = 17.82 ± 0.33 and 19.41 ± 0.24 µg/ml, respectively). The normal cell line showed a relatively high resistance toward peptide fractions (IC_50_ > 100 µg/ml) (Table 1).

**TABLE 1:** Cytotoxic activity (IC_50_ ^a^ µg/ml) of *Mucuna pruriens* peptide fractions

Peptide fraction (kDa)	SMMC‐7721	HepG2	QGY‐7703	HepG2/ADM	NIH 3T3
<1	NA^b^	NA	NA	NA	NA
1–3	NA	NA	NA	96.02 ± 1.13	NA
3–5	78.22 ± 1.30	56.80 ± 1.42	70.28 ± 1.15	81.20 ± 1.24	NA
5–10	26.31 ± 0.94	17.82 ± 0.33	19.41 ± 0.24	28.41 ± 1.04	NA
>10	57.51 ± 1.83	41.70 ± 1.02	50.03 ± 1.20	62.30 ± 1.20	NA
Cisplatin^c^	7.13 ± 0.83	4.85 ± 0.52	6.24 ± 0.94	25.61 ± 2.04	0.23 ± 0.05

^a^ Data shown as mean ± *SD* of IC_50_ values.

^b^ Not active (>100 µg/ml).

^c^ Positive control.

### 3.3 Antiviral effects of peptide fractions

The antiviral effects of different peptide fractions were evaluated against *HCV* (Table 2). IC_50_ value calculated for 5–10 kDa was significantly lower than those of peptide fractions against cell lines. The SIs of peptide fractions for all *HCV* were >3. The CC_50_ of 5–10 kDa against the cell line was 703.04 ± 5.21 µg/ml (Table 3).

**TABLE 2:** Virus inhibitions (%) of peptide fractions in different concentrations

Peptide fraction (kDa)	10 (µg/ml)	20 (µg/ml)	30 (µg/ml)	40 (µg/ml)	60 (µg/ml)	80 (µg/ml)	100 (µg/ml)
<1	10.37 ± 0.23	20.64 ± 1.65	28.61 ± 1.35	37.80 ± 2.44	43.29 ± 1.85	50.10 ± 2.72	58.50 ± 1.62
1–3	15.80 ± 0.34	27.43 ± 1.40	36.76 ± 1.70	44.90 ± 1.21	55.32 ± 2.67	69.01 ± 2.21	78.21 ± 1.45
3–5	17.11 ± 0.83	36.00 ± 0.37	49.04 ± 1.23	56.32 ± 1.80	68.72 ± 1.13	76.30 ± 1.22	88.59 ± 1.65
5–10	40.10 ± 1.25	49.05 ± 1.90	59.23 ± 1.45	66.74 ± 1.04	79.38 ± 1.22	90.01 ± 1.45	98.90 ± 1.63
>10	19.11 ± 0.45	28.33 ± 1.71	51.00 ± 1.00	59.77 ± 2.83	71.28 ± 1.83	80.10 ± 1.75	90.36 ± 1.45

**TABLE 3:** Virucidal activities of peptide fractions

Peptide fraction (kDa)	CC_50_ ^a^ (µg/ml)	IC_50_ ^b^ (µg/ml)	SI^c^
<1	259.63 ± 4.93	78.77 ± 1.62	3.30
1–3	380.72 ± 3.50	53.58 ± 2.04	7.10
3–5	533.25 ± 4.05	40.66 ± 1.40	13.11
5–10	703.04 ± 5.21	19.40 ± 2.73	36.24
>10	584.11 ± 3.60	39.61 ± 1.24	14.75

^a^ The 50% cytotoxic concentration for target cells.

^b^ The concentration of the sample required for 50% inhibition.

^c^ Selectivity index.

### 3.4 Genotoxic effects of peptide fractions

The antigenotoxic effects of the peptide fractions showed that a peptide fraction of 5–10 kDa exhibited the highest activity of protecting DNA damages. In comparison the means by Dunnett's test, with consider to the positive control, statistically significant differences can be observed among all peptide fractions with respect to tail length (*p* ≤ .05) (Table 4).

**TABLE 4:** The tail length (Mean ± *SD*) and DNA damage reduction

Peptide fraction (kDa)	Tail length (µm)	DNA Damage Reduction (%)
<1	15.84 ± 1.23	94.98
1–3	19.74 ± 1.05	91.60
3–5	70.45 ± 1.73	31.30
5–10	89.21 ± 2.04	9.43
>10	78.81 ± 1.32	21.56
Negative control	11.54 ± 0.95	–
Positive control	97.30 ± 2.23	–

## 4 DISCUSSION

Our results showed that the hydrolysis process of *M. pruriens* protein reduced the protein content of hydrolysates. It may be related to removing the insoluble portion of the hydrolysates. It was found that the fraction >10 kDa showed the highest levels of protein due to its molecular weight cut‐off. Differences among protein content of plants may be due to the different fundamental peptide, plant species, and different phase of plant maturity, growing conditions, raw material compositions, as well as the kind of the method used to obtain the protein concentrate (Yam et al., 2015). The values obtained in the present study are promising for obtaining bioactive compounds. Based on our results, we found that these peptide fractions have considerable cytotoxic effects against human hepatoma cell lines. The cytotoxic effects of proteins are depended on the fundamental peptide may be exerted by reducing the activity of membrane‐bound enzymes, disruption of cell membrane integrity via depolarization, mevalonate pathway modification of metabolism and/or apoptotic induction (Ye et al., 2018). Another exciting use of biopharmaceutical properties of some proteins is antiviral activity. Based on our antiviral assessments, some peptide fractions of *M. pruriens* inhibited *HCV*. Moreover, some peptide fractions showed high activities of protecting DNA damages. The geno‐protective effect of 5–10 kDa was considered significantly different. It is evidence that plants could offer novel alternatives to control genetic damage, existing within chronic degenerative diseases (Azizi & Fuji, 2005; Chizzola et al., 2014). It is reported that the seeds of *M. pruriens* contain an average of valuable therapeutic compounds. As these presented considerable effects, it might be employed *M. pruriens* as alternative treatments after further clinical training.

## 5 CONCLUSION

The results obtained from in vitro assays on therapeutic peptides of *M. pruriens* seeds presented considerable biological effects. The peptide fractions have been considered as candidates for the treatment of liver cancer and HCV. They were also showed high activities of protecting DNA damages. Anticancer proteins can stimulate host defense system cells via various interactions with the host's genes and proteins. This phenomenon is not only of concern to the cell biologist but also has implications with regard to scheduling of anticancer agents against human tumors. However, the available data suggest that certain therapeutic peptides may affect liver cancer and HCV, which creates the potential to alter the pharmacokinetics of co‐administered drugs. In addition, the change in exposure to peptide substrate drugs can be limited. Therefore, there is a need to better understand the clinical relevance of such interactions during drug development, to guide the safe and effective use of therapeutic peptide. These observations should be emphasized for future research on *M. pruriens* to evaluate functional roles in potential therapeutic applications of these proteins. However, as yet, the evidence available on bioactive peptides has several limitations, further studies on animals and randomized clinical trials are required to confirm these effects and enable these peptides to be used as preventive or therapeutic treatments.

## CONFLICT OF INTEREST

We declare no conflicts of interest.

## ETHICAL APPROVAL

Ethical approval for this study was obtained from Mashhad University of Medical Sciences (IR.MUMS.REC.1395.389).

## Data Availability

The data that support the findings of this study are available from the corresponding author upon reasonable request.
